# Staurosporine and NEM mainly impair WNK-SPAK/OSR1 mediated phosphorylation of KCC2 and NKCC1

**DOI:** 10.1371/journal.pone.0232967

**Published:** 2020-05-15

**Authors:** Jinwei Zhang, Antje Cordshagen, Igor Medina, Hans Gerd Nothwang, Jacek R. Wisniewski, Michael Winklhofer, Anna-Maria Hartmann

**Affiliations:** 1 Hatherly Laboratories, Medical School, College of Medicine and Health, Institute of Biomedical and Clinical Sciences, University of Exeter, Exeter, United Kingdom; 2 Xiamen Cardiovascular Hospital, School of Medicine, Xiamen University, Xiamen, China; 3 Division of Neurogenetics, School of Medicine and Health Sciences, Carl von Ossietzky University Oldenburg, Oldenburg, Germany; 4 INSERM (Institut National de la Santé et de la Recherche Médicale) Unité 1249, INMED (Institut de Neurobiologie de la Méditerranée), Aix-Marseille University UMR 1249, Marseille, France; 5 Research Center for Neurosensory Sciences, Carl von Ossietzky University Oldenburg, Oldenburg, Germany; 6 Center of Excellence Hearing4all, Carl von Ossietzky University Oldenburg, Oldenburg, Germany; 7 Department of Proteomics and Signal Transduction, Biochemical Proteomics Group, Max-Planck-Institute of Biochemistry, Martinsried, Germany; 8 Institute for Biology and Environmental Sciences IBU, Carl von Ossietzky University of Oldenburg, Oldenburg, Germany; Hopital Tenon, FRANCE

## Abstract

The pivotal role of KCC2 and NKCC1 in development and maintenance of fast inhibitory neurotransmission and their implication in severe human diseases arouse interest in posttranscriptional regulatory mechanisms such as (de)phosphorylation. Staurosporine (broad kinase inhibitor) and N-ethylmalemide (NEM) that modulate kinase and phosphatase activities enhance KCC2 and decrease NKCC1 activity. Here, we investigated the regulatory mechanism for this reciprocal regulation by mass spectrometry and immunoblot analyses using phospho-specific antibodies. Our analyses revealed that application of staurosporine or NEM dephosphorylates Thr^1007^ of KCC2, and Thr^203^, Thr^207^ and Thr^212^ of NKCC1. Dephosphorylation of Thr^1007^ of KCC2, and Thr^207^ and Thr^212^ of NKCC1 were previously demonstrated to activate KCC2 and to inactivate NKCC1. In addition, application of the two agents resulted in dephosphorylation of the T-loop and S-loop phosphorylation sites Thr^233^ and Ser^373^ of SPAK, a critical kinase in the WNK-SPAK/OSR1 signaling module mediating phosphorylation of KCC2 and NKCC1. Taken together, these results suggest that reciprocal regulation of KCC2 and NKCC1 via staurosporine and NEM is based on WNK-SPAK/OSR1 signaling. The key regulatory phospho-site Ser^940^ of KCC2 is not critically involved in the enhanced activation of KCC2 upon staurosporine and NEM treatment, as both agents have opposite effects on its phosphorylation status. Finally, NEM acts in a tissue-specific manner on Ser^940^, as shown by comparative analysis in HEK293 cells and immature cultured hippocampal neurons. In summary, our analyses identified phospho-sites that are responsive to staurosporine or NEM application. This provides important information towards a better understanding of the cooperative interactions of different phospho-sites.

## Introduction

The cation chloride cotransporter family (CCCs) consists of electroneutral secondary active cotransporters that mediate the symport of cations (Na^+^ and K^+^) coupled with chloride ions (Cl^-^) across the plasma membrane. CCCs are divided into K^+^-Cl^-^ outward cotransporters (KCC1-4), Na^+^, K^+^, Cl^-^ inward cotransporters (NKCC1-2, NCCs), the polyamine transporter CCC9, and the CCC-interacting protein CIP1 [1–3]. These transporters play a crucial role in various physiological processes like regulation of cell volume, directional ion transport across epithelial cells, secretion of K^+^, and regulation of intracellular Cl^−^concentration ([Cl^-^]_i_) in neurons [2, 4–11].

In neurons, KCC2 and NKCC1 are involved in the development and maintenance of inhibitory neurotransmission. For instance, KCC2 generates a low [Cl^-^]_i_ required for fast inhibitory neurotransmission. Binding of γ-aminobutyric acid (GABA) or glycine to their receptors, which are ligand-gated Cl^-^ channels, leads to a Cl^-^ influx and therefore hyperpolarization [8, 12]. By contrast, NKCC1 is more prevalent in immature inhibitory neurons. There, its action results in a high [Cl^-^]_i_ causing GABA or glycine to elicit a depolarizing action that opens L-type voltage-gated Ca^2+^ channels necessary for proper synapse formation [13–18].

The physiological relevance of the two cotransporters is corroborated by the phenotypes present in knockout mice. Mice with disruption of the gene *Slc12*a5 encoding both KCC2 isoforms (KCC2a and KCC2b), die shortly after birth due to motor deficits [19, 20]. Furthermore, dysfunction of KCC2 is associated with neurological and psychiatric disorders including epilepsy, neuropathic pain, spasticity, ischemic insults, brain trauma, schizophrenia, and autism [21–32]. Mice with disruption of *Slc12*a2 (NKCC1) are viable, but suffer from peripheral deafness, postnatal hyperexcitability and anti-convulsant, pain perception and male infertility [33–36]. Inhibition of KCC2 promotes formation of pathological conditions whereas inhibition of NKCC1 seems to prevent or at least alleviate pathological phenotypes [37]. This renders these two transporters a prime pharmacotherapeutic target and fosters interest in understanding posttranslational mechanisms of their regulation [12, 38–40]. The main focus is set on phospho-related mechanisms. The broad kinase inhibitor staurosporine was used to enhance KCC2 transport activity in immature auditory brainstem [41] and hippocampal cultured neurons [42], and to decrease NKCC1 activity [43–46]. Furthermore, N-ethylmaleimide (NEM), that likely modulates the activity of kinases and phosphatases, activates KCC2 [47–50] and inhibits NKCC1 [45, 51]. Thus, both compounds reciprocally regulate KCC2 (activation) and NKCC1 (inhibition). Understanding their mode of action provides the basis for further pharmacological studies and important insight into the molecular mechanisms involved in physiological regulation of these two transporters.

Since KCC2 and NKCC1 are present in the same neuronal populations but transport K^+^ and Cl^-^ in opposite direction [52–54], regulatory mechanisms are required to coordinate their activity [4, 38, 55–60]. Indeed, several kinases and phosphatases regulate their transport activity. *In vitro* and *in vivo* analyses revealed that members of the with-no-lysine kinase (WNKs) family in combination with their downstream targets STE20/SPS1-related proline/alanine rich kinase (SPAK) and oxidative stress response kinase (OSR1) are the most prominent kinases that regulate KCC2 and NKCC1 activity in a reciprocal way. SPAK/OSR1, activated by WNK1, phosphorylate Thr^6^ and Thr^1007^ of KCC2 [48, 59, 61–64]. WNKs also interact with a yet unknown kinase to phosphorylate Thr^906^ of KCC2 [48, 63]. Site-directed mutagenesis studies indicate that simultaneous mutation of Thr^906^ and Thr^1007^ to alanine (mimicking the dephosphorylated state) activates KCC2 [61, 65–67]. WNK/SPAK/OSR1 also phosphorylates the N-terminal residues Thr^203^, Thr^207^, Thr^212^ and Thr^217^ of NKCC1 [38, 59, 60, 68–76] thereby activating NKCC1 [72, 75]. Thus dephosphorylation (KCC2) and phosphorylation (NKCC1) of WNK/SPAK/OSR1 specific phospho-sites enhance KCC2 and NKCC1 activity.

Phosphorylation can also enhance KCC2 activity. Phosphorylation of Ser^932^, Thr^934^, Ser^937^, or the protein kinase C (PKC) site Ser^940^, all residing in exon 22, increases its transport activity [47, 68, 77]. The multiplicity of phosphorylation/dephosphorylation sites on KCC2 offers a complex toolbox to gradually fine-regulate its activity and integrate different signaling pathways [4, 40, 65, 68].

Initially, both staurosporine and NEM were thought to act through a similar mechanism [47], but recent findings revealed that they act differentially on specific KCC2 phospho-sites [40]. Furthermore, staurosporine and NEM mediated effects involve both KCC2 phosphorylation and dephosphorylation [40, 48]. To gain insight into their mode of action, we analyzed the impact of these compounds on phosphorylation of specific KCC2 and NKCC1 phospho-sites using large-scale phosphoproteomics studies and phospho-site specific antibodies in stably transfected HEK293 cells and immature primary cultures of hippocampal neurons.

## Material and methods

### Cell culturing of HEK293 cells

For K^+^-Cl^-^ cotransporter activity measurements, stably transfected rat KCC2b HEK293 cells (HEK^*rn*KCC2b^) [55] were plated in a 0.1 mg/ml poly-L-lysine coated black-well 96 well culture dish (Greiner Bio-One, Frickenhausen, Germany) at a concentration of 100,000 cells/well. The remaining cells were plated on a 0.1 mg/ml poly-L-lysine-coated class coverslip and ~18 h later analyzed by immunocytochemistry.

### Primary cultures of rat hippocampal neuron

All animal procedures were carried out in accordance with the European Union Directive of 22 September (2010/63/EU). The protocol was approved by the INSERM Local committee (Number 0287.01, delivered by Ministère de l’Education et de la Recherche). Hippocampi from 18-day-old rat embryos were dissected and dissociated using trypsin (0.05%) and plated in 60-mm culture dishes at a density of 100,000 cells cm^−2^ in minimal essential medium (MEM) supplemented with 10% NU serum (BD Biosciences, Le Pont de Claix, France), 10 mM glucose, 1 mM sodium pyruvate, 2 mM glutamine, and 100 U ml^−1^ penicillin–streptomycin as previously described [78]. On day 7 of culture incubation, half of the medium was changed to MEM with 2% B27 supplement (Invitrogen). 24 h prior to plating, dishes were coated with poly-ethylenimine (5 μg/ml).

### Immunocytochemistry

For immunocytochemistry, all steps were performed at room temperature. HEK293 cells grown on poly-L-lysine-coated coverslips were fixated for 10 min with 4% paraformaldehyde in 0.2 M phosphate buffer (PBS). After three washing steps in PBS, cells were incubated with blocking solution (2% bovine serum albumin, 10% goat serum in PBS) for 30 min. Primary antibody solution (anti-KCC2 N1-12; 1:1,000; Neuromab, Carlifornia, USA) was added in carrier solution (0.3% Triton X-100, 1% bovine serum albumin, 1% goat serum in PBS) for 1 h. After washing three times in PBS, the secondary antibody, which was conjugated to a fluorescent probe (Alexa Fluor 488 goat anti-mouse; 1:1,000; Thermo Fisher Scientific, Bremen, Germany) was incubated for 1 h. After three washes in PBS, cells were mounted onto glass slides with Mowiol (Roth, Karlsruhe, Germany) and DAPI (Roth; 1:1,000). Photomicrographs were taken using Biozero BZ-8100E (Keyence, Neu-Isenburg Deutschland) fluorescence microscope (Leica, Wetzlar, Germany).

### Determination of K^+^-Cl^-^ cotransport

KCC2 transport activity was determined by Cl^−^dependent uptake of Tl^+^ in HEK293 cells as previously described [47, 79, 80]. To initiate the flux measurement, the medium in the 96-well culture dish was replaced by 80 μl preincubation buffer (100 mM N-methyl-D-glucamine-chloride, 5 mM HEPES, 5 mM KCl, 2 mM CaCl_2_, 0.8 mM MgSO_4_, 5 mM glucose, pH 7.4, 160 mmol / kg ± 2,04 mmol/kg) supplemented with 2 μM FlouZin-2 AM dye (Thermo Scientific, Bremen, Germany) plus 0.2% (wt/vol) Pluronic F-127 (Thermo Scientific, Bremen, Germany) and incubated for 48 min at room temperature. Cells were then washed 3 times with 80 μl preincubation buffer and incubated for 15 min with 80 μl preincubation buffer plus 0.1 mM ouabain to block Na^+^/K^+^ ATPase activity. Three technical and five biological replicates were performed for each construct. Afterwards, the 96-well plate was placed into a fluorometer (Fluoroskan Accent, Thermo Scientific, Bremen, Germany) and each well was injected with 40 μl 5 x Tl^+^ stimulation buffer (12 mM Tl_2_SO_4_, 100 mM NMDG, 5 mM Hepes, 2 mM KCl, 2 mM CaCl_2_, 0.8 mM MgSO_4_, 5 mM glucose, pH 7.4; 175 mmol/kg ± 2 mmol/kg). Fluorescence was determined in a kinetic dependent manner (excitation 485 nm, emission 538 nm, 1 frame in 5 s in a 200 s time span) across the entire cell population in a single well. By using linear regression of the initial values of the slope of Tl^+^-stimulated fluorescence increase, transport activity was calculated [79, 80].

To determine the dose-response profile, increasing concentration of staurosporine (8–80 μM) and NEM (25–3000 μM) were applied to the preincubation buffer 15 min prior flux measurement. This was done for three technical replicates and at least five independent measurements were performed.

### Statistical analyses

**Fig 1:**
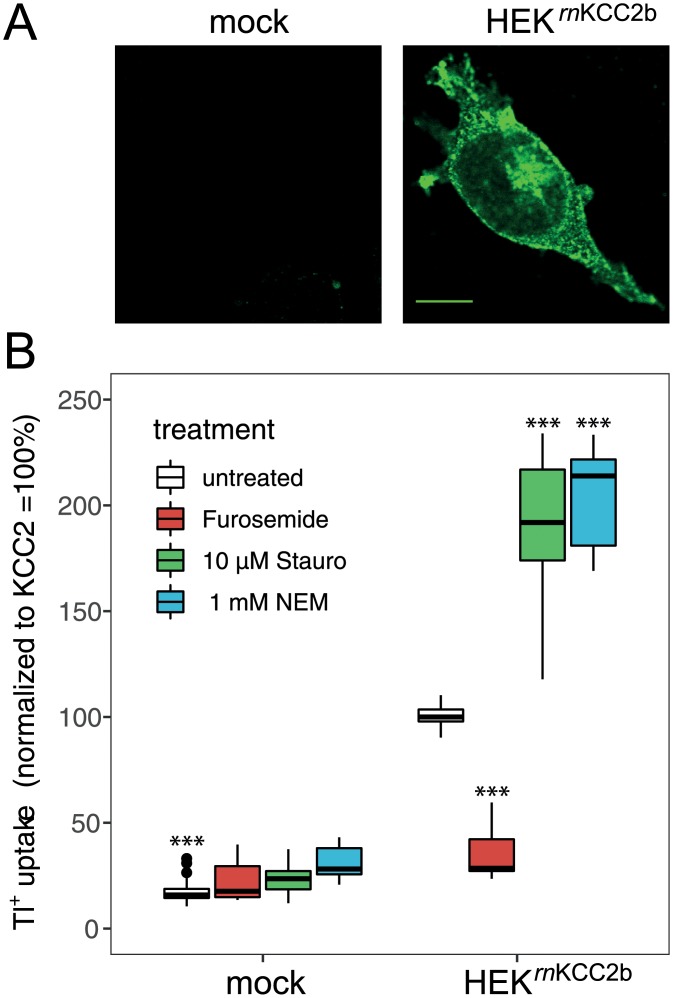
Staurosporine and NEM increase KCC2 activity. A: Immunoreactivity of stably transfected HEK^*rn*KCC2b^ cells was detected with labeling at the cell membrane and cytosol. Photomicrographs were taken with a confocal laser-scanning microscope. Scale bar, 10 μm. B: Transport activity of *rn*KCC2b was determined by Tl^+^ flux measurements. Treatment with 10 μM staurosporine or 1 mM NEM resulted in an approximately twofold increase of *rn*KCC2b activity. Application of the loop diuretics furosemide (2 mM) significantly inhibited *rn*KCC2b function. The graph represents the data of at least five independent measurements (each consisting of three technical replicates) normalized to *rn*KCC2b. Statistical analyses: ***, *p* < 0.001 versus HEK^*rn*KCC2b^; Error bars represent SD.

The majority of data populations illustrated in Figs 1, 3 and 4 showed non-normal distributions (verified using Shapiro-Wilk normality test at 0.05 significance level). Therefore, a nonparametric test (Wilcoxon-Mann-Whitney rank sum test) was employed for the comparison between different groups of data. The resulting *p*-values were adjusted with the Bonferroni correction for multiple testing. Note that only *p*-values < 0.01 were considered to reduce the chances of false positives (type I errors). The sample size n always refers to the number of biological replicates (independent preparations). The activity of each independent preparation was determined as mean over three technical replicates. The average scatter within technical replicates was 3 times smaller than the scatter across biological replicates for a given treatment.

For dose-response analyses, we used the nls function from the stats package in R (version 3.5.1) to model the data points with the Hill-Langmuir equation,

$$y(x;{\text{ED}}_{50},n,y_{\text{sat}})=y_{0}+y_{\text{sat}}\frac{x^{n}}{{{ED}_{50}}^{n}+x^{n}},$$ (1)

where *y* is the Tl^+^ uptake of HEK^*rn*KCC2b^ cells, *y*_0_ is the Tl^+^ independent-baseline activity (*y*_0_ = 100%), *y*_sat_ is the maximum activity (equivalent to V_max_ in reaction rate equation) relative to baseline, *x* is the concentration of the agonist, *n* is the Hill coefficient, and ED_50_ is the agonist concentration that produces 50% of the saturation response. The 95% confidence intervals for the dose response curves were determined with the function NonlinearModelFit [“MeanPredictionBands”] in Wolfram Mathematica. For the boxplots, the box extends from the upper (Q1) to the lower (Q3) quartile. The line inside the box represents the median. The whiskers extend to the outermost data point that falls within upper inner and lower inner Quartile fences [Q1+1.5(IQR)] and [Q3-1.5(IQR)], respectively, where IQR = Q1-Q3 is the interquartile range.

### Treatment of stably transfected HEK^rnKCC2b^ cells and hippocampal neurons

For mass spectrometry and immunoblot analyses, stably transfected HEK^*rn*KCC2b^ cells [55] were grown in 75 cm² cell-culture flask. Cells were washed with 5 ml flux hypotonic preincubation buffer (100 mM N-methyl-D-glucamine-chloride, 5 mM HEPES, 5 mM KCl, 2 mM CaCl_2_, 0.8 mM MgSO_4_, 5 mM glucose, pH 7.4; 160 mmol/kg±2,04 mmol/kg) and then treated with or without 8 μM staurosporine or 1 mM NEM. Cells were centrifuged at 500 rpm for 3 min and the resulting pellets used for immunoblot analyses or mass spectrometry analyses.

For immunoblot analyses of immature (9 days *in vitro*, (DIV)) rat hippocampal neurons, half of the media from culture dishes containing neurons (2.5 ml from each dish) was collected to prepare samples including 16 μM staurosporine (from 10 mM stock solution in DMSO), 1 mM NEM (by direct dissolving of the NEM powder) or DMSO (vehicle, same volume as for staurosporine), respectively. The samples were distributed dropwise into the culture dishes that gave final concentration of 8 μM staurosporine or 0.5 mM NEM and cultures were incubated 15 min at 37 °C (5% CO_2_). After incubation, neurons were rinsed twice with Hanks’ Balanced Salt solution (HBSS, ThermoFisher Scientific) precooled to 4 °C. The rinsage solution was replaced with ice-cold HBSS containing a cocktail of protease and phosphatase inhibitors (ThermoFisher Scientific) and neurons rapidly scraped and centrifuged at 4 °C (10,000 g, 3 min). Pellets were frozen in liquid nitrogen and kept for future analyses at -80 °C.

### Multi-Enzyme Digestion Filter Aided Sample preparation (MED FAS)

For mass spectrometry analyses, cell pellets of untreated, staurosporine or NEM treated HEK^*rn*KCC2b^ cells were lysed in 2% SDS in 0.1 M Tris-HCl, pH 8.0, containing 0.1 M DTT as described previously [81]. Total protein was determined using WF-assay in micro-titer plate format [82]. Aliquots of the cell lysate containing 1–2 mg were processed in Amicon Ultra 15 Ultracel 30k (Merck Millipore, Darmstadt) devices as described previously [83] with several modifications using the MED FASP method [84]. Briefly, SDS and other low molecular weight material were removed by centrifugation at 4,000 x *g* in 5 consecutive washes with UA buffer containing 8 M urea (ultrapure, Merck, Darmstadt) in 0.1 M Tris pH 8. Following two washes with 5 ml of 0.1M Tris-HCl, pH 8 (DB buffer), 20 μg of LysC (Wako, Neuss) in 0.5 mL of DB was added to the filter. Samples were digested overnight at 37 °C and peptides were collected by centrifugation. Next, the material retained in the filter was cleaved with 10 μg trypsin in 0.5 ml DB at 37 °C for 4 h and the peptides were eluted as previously. To increase the yield of peptides, filters were washed twice with 0.5 mL DB. Concentration of peptides was determined by WF-assay [82].

### TiO_2_-based enrichment of phosphopeptides

Phosphopeptides were enriched using TiO_2_-beads [85] with several modifications [83]. Briefly, 25 mg of ‘Titansphere TiO_2_ 10 μm’ (GL Sciences, Inc., Japan) were suspended in 50 μl of 3% (m/v) dihydroxybenzoic acid in 80% (v/v) CH_3_CN, 0.1% CF_3_COOH and diluted 1:4 with water before use. Ten microliters of this slurry (1 mg beads) were added and samples incubated under continuous agitation for 20 min. The mass ratio of the beads and peptides was 3:1. Then, the titanium beads were sedimented by centrifugation at 5,000 x *g* for 1 min and the supernatants were collected and mixed with another portion of the beads and incubated as above. The bead-pellets were resuspended in 150 μl of 30% (v/v) CH_3_CN containing 3% (v/v) CF_3_COOH and transferred to a 200 μl pipet tip plugged with one layer of glass microfiber filter GFA (Whatman). The beads were washed three times with 30% (v/v) CH_3_CN, 3% CF_3_COOH (v/v) solution and three times with 80% CH_3_CN (v/v), 0.3% CF_3_COOH (v/v) solution. Finally, the peptides were eluted from the beads with 100 μL of 40% CH_3_CN (v/v) containing 15% NH_4_OH (m/v) and were vacuum-concentrated to ∼4 μl.

### Mass-spectrometric analysis

Analysis of peptide mixtures was conducted using a QExactive HF-X mass spectrometer (Thermo-Fisher Scientific, Palo Alto). Aliquots containing <1 μg of total peptide were chromatographed on a 50 cm column with 75 μm inner diameter packed C_18_ material. Peptide separation occurred at 300 nl/min for 95 min using an acetonitrile gradient of 5–30%. The temperature of the column oven was 55°C. The mass spectrometer operated in data-dependent mode with survey scans acquired at a resolution of 60,000. Up to the top 15 most abundant isotope patterns with charge ≥ +2 from the survey scan (300–1650 m/z) were selected with an isolation window of 1.4 m/z and fragmented by HCD with normalized collision energies of 25. The maximum ion injection times for the survey scan and the MS/MS scans were 20 and 28 ms, respectively. The ion target value for MS1 and MS2 scan modes was set to 3×10^6^ and 10^5^, respectively. The dynamic exclusion was 30 s. The MS spectra were searched using MaxQuant software. A maximum of two missed cleavages was allowed. The maximum false peptide and protein discovery rate was specified as 0.01. The whole mass spectrometry analyses were performed in three technical and two biological replicas per treatment.

### Immunoblot and phospho-antibody immunoprecipitation analyses

Lysate protein samples were subjected to immunoblot and immunoprecipitation as previously described [63]. Protein samples (40 μg) were boiled in sample buffer for 5 min, resolved by 7.5% sodium dodecyl sulfate polyacrylamide-gel electrophoresis and electrotransferred onto a polyvinylidene difluoride membrane. Membranes were incubated for 30 min with TBST (Tris-buffered saline, 0.05% Tween-20) containing 5% (w/v) skim milk. Blots were then washed six times with TBST and incubated for 1 h at room temperature with secondary HRP-conjugated antibodies diluted 5000-fold in 5% (w/v) skim milk in TBST. After repeating the washing steps, signals were detected with enhanced chemiluminescence reagent. Immunoblots were developed using ChemiDoc^™^ Imaging Systems (Bio-Rad, Feldkirchen). Figures were generated using Photoshop/Illustrator (Adobe). Band densities of bands were measured with ImageJ. Calculation of intensity ratios is based on (phospho-dimeric KCC2 + phospho-monomeric KCC2) / (total dimeric KCC2 + total monomeric KCC2), (total dimeric KCC2 + total monomeric KCC2)/β-actin or tubulin, SPAK pThr^233^/SPAK, SPAK pSer^373^/SPAK, SPAK/β-actin or tubulin, NKCC1 pThr^203,207,212^/NKCC1, NKCC1/β-actin or tubulin, as reported previously [66]. Mann–Whitney U-test was used for comparison between 2 independent groups and Wilcoxon matched pairs test was employed to compare paired data (GraphPad Prism 7.0, San Diego, CA, USA).

For phospho-antibody immunoprecipitation, KCC isoforms were immunoprecipitated from indicated cell extracts. 2 mg of the indicated clarified cell extract was mixed with 15 μg of the indicated phospho-specific KCC antibody conjugated to 15 μl of protein-G–Sepharose, in the added presence of 20 μg of the dephosphorylated form of the phosphopeptide antigen, and incubated 2 hours at 4 °C with gentle shaking. Immunoprecipitates were washed three times with 1 ml of lysis buffer containing 0.15 M NaCl and twice with 1 ml of buffer A. Bound proteins were eluted with 1x LDS sample buffer.

### Antibodies

The following antibodies were purchased from Division of Signal Transduction Therapy Unit at the University of Dundee: KCC2A phospho-Thr^906^ (SAYTYER(T)LMMEQRSRR [residues 975–989 of human KCC3A] corresponding to SAYTYEK(T)LVMEQRSQI [residues 899–915 of human KCC2A], S959C); KCC2A phospho-Thr^1007^ (CYQEKVHM(T)WTKDKYM [residues 1032–1046 of human KCC3A] corresponding to TDPEKVHL(T)WTKDKSVA [residues 998–1014 of human KCC2A], S961C); SPAK/OSR1 (T-loop) phospho-Thr^233^/Thr^185^ antibody (226–238 of human SPAK or residues 178–190 of human OSR1, TRNKVRKpTFVGTP, S204C); SPAK/OSR1 (S-motif) phospho-Ser^373^/Ser^325^ antibody (367–379 of human SPAK, RRVPGSpSGHLHKT, which is highly similar to residues 319–331 of human OSR1 in which the sequence is RRVPGSpSGRLHKT, S670B); SPAK (427–443 of human SPAK, QSLSVHDSQGPPVGTP, S849C); NKCC1 phospho-Thr203+Thr207+Thr212 (residues 198–217 of human NKCC1, HYYYDpTHTNpTYYLRpTFGHNT, S763B); NKCC1 phospho-Thr^212^/Thr^217^ (residues 208–223 of human NKCC1, YYLRpTFGHNpTMDAVPR, S063D); NKCC1-total antibody (residues 1–260 of shark NKCC1, S841B). Pan-KCC2 antibody (residues 932–1043 of human KCC2) was from NeuroMab (73–013). KCC2 phospho-Ser940 antibody was from ThermoFisher Scientific (PA5-95678). PKCδ phospho-Thr^505^ antibody (9374) and β-Actin (D6A8) antibody (12620) were from Cell Signaling Technology. Anti (neuronal)-β-Tubulin III antibody was from Sigma-Aldrich (T8578). Secondary antibodies coupled to horseradish peroxidase used for immunoblotting were obtained from Pierce. IgG used in control immunoprecipitation experiments was affinity-purified from pre-immune serum using Protein G-Sepharose.

## Results

### Dose-response analyses of staurosporine and NEM in stably transfected HEK^rnKCC2b^ cells

Staurosporine and NEM generally activate KCCs [40, 47, 86, 87]. To closer characterize their mode of action, we used stably transfected HEK^*rn*KCC2b^ cells, as high amount of the cotransporter is advantageous for subsequent biochemical analyses. Immunoreactivity of HEK^*rn*KCC2b^ cells against KCC2 was detected in all cells with clear labeling at the cell membrane and cytosol (Fig 1A). This is in agreement with previous cell surface expression analyses that detected 11.8 ± 1.4% of total KCC2 in stably transfected *rn*KCC2b at the cell surface [79].

To quantify the impact of staurosporine and NEM on *rn*KCC2b transport activity, Tl^+^ flux measurements were performed [79, 80, 88]. Transport activity in HEK^*rn*KCC2b^ cells was 5.6 times higher compared to background (HEK^*rn*KCC2b^ 100% ± 5.32%; mock; 17.7% ± 5.5%; *p* = 4.3x10^-5^; n = 5) (Fig 1B). Here and elsewhere in the text, numbers indicate mean +/- SD, *p* was determined using Wilcoxon-Mann-Whitney rank sum test. Treatment with 10 μM staurosporine or 1 mM NEM resulted in an approximately twofold increase of *rn*KCC2b activity compared to untreated HEK^*rn*KCC2b^ cells (staurosporine: 187.5% ± 37.1%, *p* = 3x10^-3^; NEM: 204% ± 23.2%; *p* = 3x10^-3^). The loop diuretics furosemide, that specifically inhibits the function of cation chloride cotransporters [89, 90], significantly inhibited the function of *rn*KCC2b (34.7 ± 12.2%, *p* = 4 x10^-5^).

**Fig 2:**
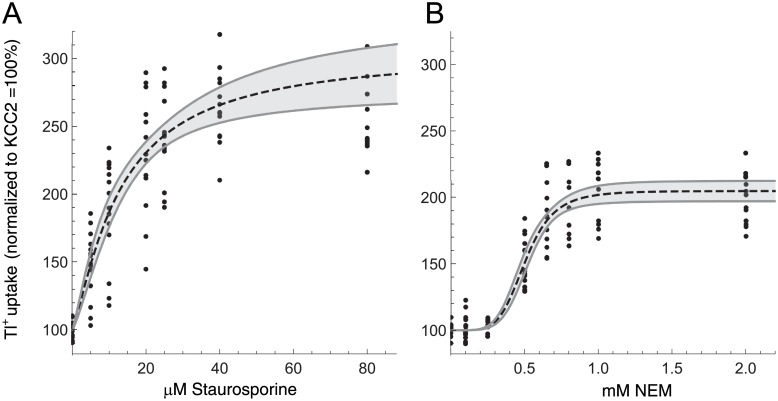
Dose-response relationship of staurosporine and NEM on KCC2 transport activity. HEK^*rn*KCC2b^ cells were treated with different concentrations of staurosporine (5–80 μM; A) or NEM (25–3,000 μM; B) for 15 min. Transport activity of *rn*KCC2b was determined by Tl^+^ flux measurements. Treatment with staurosporine resulted in a classical Michaelis-Menden curve, whereas NEM treatment showed a sigmoidal curve with a Hill coefficient of 5.2 ± 1.3 indicating a positive cooperative effect on *rn*KCC2b. The graph represents the data of at least five independent measurements (each consisting of three technical replicates) normalized to untreated HEK^*rn*KCC2b^.

Next, we determined the dose-response relationships of staurosporine and NEM on *rn*KCC2b transport activity by treating HEK^*rn*KCC2b^ cells with different concentrations of staurosporine (5–80 μM) or NEM (25–3,000 μM). Fig 2 represents the dose-response curve for both agents. The dose-response curve for staurosporine (Fig 2A) was approximately a rectangular hyperbola (*n* ≈ 1, not significantly different from unity). This reflects Michaelis-Menten kinetics and suggests absence of cooperative effects. In contrast, the dose response curve for NEM (Fig 2B) had a pronounced sigmoidal shape (*n* ≈ 5), which reflects cooperative binding kinetics. The effective dose ED_50_ (representing the potency) for staurosporine of stably transfected HEK^*rn*KCC2b^ was 12.8 ± 4.9 μM and the maximal efficacy (E_max_) was 205 ± 40%. The ED_50_ value for NEM was 0.5 ± 1.3 mM and E_max_ was 105 ± 6%. For further analyses, we used a concentration of 8 μM staurosporine and 1 mM NEM, if not indicated otherwise as these concentrations significantly increase KCC2b transport activity.

### Identification of CCC phosphorylation sites in stably transfected HEK^rnKCC2b^ cells by mass spectrometry analyses upon treatment with staurosporine or NEM

Phosphoproteomics by mass spectrometry has the advantage of providing an unbiased survey of phospho-sites. Therefore, we here used for the first time this technique to gain insight on the impact of staurosporine and NEM on *rn*KCC2b phospho-sites in HEK^*rn*KCC2b^. For each condition, two biological and three technical replicas were performed. Phospho-sites detected in at least one technical experiment are listed in Table 1. We first mapped KCC2b phospho-sites in untreated HEK^*rn*KCC2b^ cells. Twelve phospho-sites were identified: Ser^25^, Ser^26^, Ser^31^, Thr^32^, Thr^34^ in the cytoplasmic N-terminus and Thr^906^, Ser^937^, Ser^940^, Thr^1007^, Ser^1022^, Ser^1025^, and Ser^1026^ in the C-terminus (Table 1, untreated). These sites include all phospho-sites already present in PhosphositePlus (Table 2) [91]. Recently phosphoproteomic data deposited in Phosida and Phosphosite plus revealed that rat KCC2b tissue only harbors seven phospho-sites (Table 2). Thus, we here report five phospho-sites (Ser^25^, Thr^32^, Thr^906^, Ser^937^ and Thr^1007^) that were so far only reported for mouse but not rat KCC2 tissue (Table 2). These sites most likely reflect different expression of kinases and phosphatases in different tissues (HEK293 vs. rat brain tissue) [69] or increased detection rate in stably transfected HEK^*rn*KCC2b^ cells.

**Table 1:** Phospho-sites of stably transfected HEK^rnKCC2b^ cells. Stably transfected HEK^*rn*KCC2b^ were treated with or without 1 mM NEM or 8 μM staurosporine before they were analyzed by mass spectrometry. The protein accession numbers are: *hs*NKCC1 (P55011), *hs*KCC1 (Q9UP95), *rn*KCC2b (Q9H2X9-2), *hs*KCC4 (Q9Y666).

Sample	Phosphorylation sites	Phosphopeptides	untreated	NEM treated	staurosporine treated
***hs*NKCC1**	T212; T217	**T**FGHN**T**MDAVPR	(2)		
	S242	LLRP**S**LAELHDELEK	(1)	(1)	
	T266	EPFEDGFANGEES**T**PTR	(3)	(2)	(2)
	S940	EGLDISHLQGQEELLS**S**QEK	(2) (2)	(2) (1)	(3) (1)
	Y956; S957	DVVVSVE**YS**KK	(3) (2)	(3) (1)	(3) (3)
***hs*KCC1**	T983	IQM**T**WTRDK	(2)		
***rn*KCC2b**	S25; S26	E**SS**PFINSTDTEK	(1)		
	S26	ES**S**PFINSTDTEK	(2) (3)	(3) (3)	(3) (3)
	S31	ESSPFIN**S**TDTEK	(2)	(1)(1)	
	T32	ESSPFINS**T**DTEK	(1)	(1)	(1)
	T34	ESSPFINSTD**T**EK	(3) (2)	(2)	(1)
	T906	**T**LVMEQRSQILK	(3)	(3)	(3)
	S937	EIQSITDE**S**RGSIR	(2)		
	S940	EIQSITDESRG**S**IR	(2) (3)	(2)	(1)
	T1007	VHL**T**WTK	(3)	(3)	(3)
	S1022; S1025	GP**S**PV**S**SEGIK	(3) (1)	(3)	(3) (1)
	S1022; S1025; S1026	GP**S**PV**SS**EGIK	(1)	(1)	
	S1022; S1026	GP**S**PVS**S**EGIK	(3)	(3)	(3)
	S1026	GPSPVS**S**EGIK	(3)	(3)	(3)
***hs*KCC4**	T926	**T**LMMEQR	(2)		
	T980	VQM**T**WTREK	(3)	(1)	

The number in brackets indicates in how many technical replica a given phospho-site was detected (max. 3). Each bracket provides the results of one biological experiment.

**Table 2:** Phospho-sites in PhosphoSitePlus and PHOSIDA detected by mass spectrometry analyses.

Phosphosite Plus *rn*KCC2b	Phosphosite Plus *hs*KCC2b	Phosphosite Plus *mm*KCC2b	Phosida *mm*KCC2b	Transport activity measured by:
-	-	**S**^**25**^	**S**^**25**^	[47, 65]
**S**^**26**^	**-**	**S**^**26**^	**S**^**26**^	[47, 65]
**S**^**31**^	**S**^**31**^	**-**	**S**^**31**^	(40)
-	-	-	**(T**^**32**^**)**	-
**T**^**34**^	**T**^**34**^	**T**^**34**^	**T**^**34**^	[40, 92]
-	Y^594^	-	-	-
-	S^599^	-	-	-
-	-	-	S^649^	-
-	T^798^	-	-	-
-	T^808^	-	-	-
-	-	**T**^**906**^	**T**^**906**^	[47, 61, 66]
-	-	S^913^	-	-
-	-	S^932^	(S^932^)	[40]
-	-	-	(T^934^)	[47]
-	-	**S**^**937**^	**S**^**937**^	[47]
**S**^**940**^	-	**S**^**940**^	**S**^**940**^	[48, 77]
-	-	S^988^	-	-
-	-	T^999^	-	[40]
-	-	**T**^**1006**^	**T**^**1006**^	[47, 48, 61, 66]
-	-	-	T^1008^	[40]
**S**^**1022**^	-	**S**^**1021**^	**S**^**1021**^	[47]
**S**^**1025**^	-	**S**^**1024**^	**(S**^**1024**^**)**	[47]
**S**^**1026**^	-	**S**^**1025**^	**S**^**1025**^	[47]
-	-	S^1044^	-	-

Abbreviations used are as follows: *rn*, *Rattus norvegicus*; *hs*, *Homo sapiens*; *mm*, *Mus musculus*. Phospho-sites detected in the present mass spectrometry study are marked in bold.

As KCC1, KCC4, and NKCC1 are endogenously expressed in HEK293 cells and as mass spectrometric analysis provides data on most proteins in a given sample, we also investigated phosphorylation sites of the following proteins: *hs*KCC1 (Thr^893^, analogous to *rn*KCC2b Thr^1007^), *hs*KCC4 (Thr^926^ and Thr^980^, analogous to *rn*KCC2b Thr^906^ and Thr^1007^) and *hs*NKCC1 (Thr^212^, Thr^217^, Ser^242^, Thr^266^, Thr^268^, Ser^940^, Tyr^956^, and Ser^957^). These phospho-sites were already previously deposited in PhosphositePlus and Phosida (S1–S3 Tables). Overall, we detected only a low proportion of all so far deposited phospho-sites for these three cotransporters. This might reflect low expression levels in HEK293 cells.

Next, we investigated the phosphorylation pattern of *rn*KCC2b upon staurosporine and NEM treatment. Phosphorylation at Ser^26^, Thr^32^, Thr^34^, Thr^906^, Ser^940^, Thr^1007^, Ser^1022^, Ser^1025^, Ser^1026^ of *rn*KCC2b were still present upon treatment with staurosporine or NEM, whereas phosphorylation of Ser^25^ and Ser^937^ was not detected anymore in either of the two conditions (Table 1). Additionally, no phosphorylation was detected for Ser^31^ after treatment with staurosporine.

Regarding endogenously expressed CCCs, the phospho-site Thr^983^ of *hs*KCC1 (analogous to *rn*KCC2b Thr^1007^), Thr^926^ of *hs*KCC4 (analogous to *rn*KCC2b Thr^906^) and Thr^212/217^ and Thr^266/268^ of *hs*NKCC1 could not be detected upon application of either of the two reagents. Additionally, the phospho-sites Thr^980^ of *hs*KCC4 (analogous to *rn*KCC2b Thr^906^) and Ser^242^ of *hs*NKCC1 were not detected anymore after staurosporine treatment. The phospho-sites Thr^266^, Ser^940^ and Tyr^956^/Ser^957^ of *hs*NKCC1 were still present upon treatment with staurosporine and NEM.

Several kinases were described to directly phosphorylate KCC2 and NKCC1. This includes kinases of the WNK-SPAK/OSR1 and PKC mediated phosphorylation pathways. To gain further insight into the regulatory phosphorylation mechanism, we explored their phosphorylation pattern as well. We detected several phosphorylation sites in *hs*WNK1, *hs*WNK2, and *hs*SPAK [69, 93, 94] (Table 3). Upon all, we observed phosphorylation of the activating T-loop residue Ser^382^ of *hs*WNK1 and the S-loop phosphorylation site of Ser^372/373^ of *hs*SPAK that is phosphorylated by WNK1 [63, 69, 93]. Upon treatment with staurosporine or NEM, Ser^382^ of *hs*WNK1 and Ser^372/373^ of *hs*SPAK were not detected anymore (Table 4). We were not able to detect phosphorylation of Thr^233^ that is located in the T-loop kinase domain of *hs*SPAK. Normally, this site is directly phosphorylated by WNK1 and WNK4 to activate SPAK [60, 63, 69, 95]. Furthermore, we did not detect any phospho-sites of the ubiquitously expressed WNK3 and WNK4. Again, this probably results from low expression rates in HEK293 cells.

**Table 3:** Phospho-sites of *hs*SPAK, *hs*WNK1 and *hs*WNK2 endogenously expressed in stably transfected HEK^rnKCC2b^ cells. Stably transfected HEK^*rn*KCC2b^ were treated with or without 1 mM NEM or 8 μM staurosporine before they were analyzed by mass spectrometry. The protein accession numbers are: *hs*SPAK (AAC72238.1), *hs*WNK1 (Q9H4A3.2), *hsWNK2* (Q9Y3S1.4).

sample	Phosphorylation site	Phosphopeptides	untreated	staurosporine treated	NEM treated
***hs*SPAK**	308	EMMKK**Y**GK	(2) (1)	(1)	(2)
	354	LL**T**RTPDIAQRAK	(1)	(2)	(1)
	356	LLTR**T**PDIAQRAK	(2)	(2)	(1)
	403	AAF**S**QEK	(3)	(1)	(1)
	518; 520	ALK**T**L**T**FK	(3) (2)	(2) (3)	(3) (3)
	372; 373	VRRVPG**SS**GHLHK	(1)		
***hs*WNK1**	11	QS**S**TPGSLFLSPPAPAPK	(2)	(1)	
	167; 170	DRPV**S**QP**S**LVGSK	(3)	(2)	(3)
	167; 174	DRPV**S**QPSLVG**S**K	(3)	(3)	(2)
	170	DRPVSQP**S**LVGSK	(3)		(2)
	174	DRPVSQPSLVG**S**K	(3) (3)	(3) (3)	(2) (3)
	183	EEPPPAR**S**GSGGGSAK		(1)	(1)
	185	EEPPPARSG**S**GGGSAK		(1)	
	183; 185	EEPPPAR**S**G**S**GGGSAK	(3)	(3)	(3)
	382	**S**VIGTPEFMAPEMYEEK	(1)		
	599	QQVEQSSA**S**QTGIK	(2) (3)	(3) (3)	(2)
	1220	DDYGFSG**S**QK	(2)	(1)	
	1849	EGPVLAT**S**SGAGVFK	(3)	(1)	
	1978	EGPVA**S**PPFMDLEQAVLPAVIPK	(3)	(3)	(3)
	2121	VPPAVIIPPAAPL**S**GR	(2)		(1)
	2245	G**T**FTDDLHK	(1)		
	2372	SI**S**NPPGSNLRTT	(3) (3)	(3) (3)	(3)
***hs*WNK2**	560	EQQDVG**S**PDK	(2)	(3)	(3)
	1889	AG**S**LGPETPSR	(3)	(3)	(3)
	1862	QA**S**LPVSGSVAGDFVK	(1) (3)		(2) (1)

The number in brackets indicates in how many technical replica a given phospho-site was detected (max. 3). Each bracket provides the results of one biological experiment.

**Table 4:** Phospho-sites of *hs*PKC endogenously expressed in stably transfected HEK^rnKCC2b^ cells. Stably transfected HEK^*rn*KCC2b^ were treated with or without 1 mM NEM or 8 μM staurosporine before they were analyzed by mass spectrometry. The protein accession numbers are: *hs*PKC Alpha (P17252), *hs*PKC Beta (P05771-2), *hs*PKC Delta (Q05655), *hs*PKC Epsilon (Q02156), *hs*PKC Theta (Q04759), *hs*PKC Eta (P24723), and *hs*PKC Iota (P41743).

classes	sample	Phosphorylation site	Phosphopeptides	untreated	staurosporine treated	NEM treated
conventional	**PKC Alpha**	10;11	ADVFPGND**ST**ASQDVANRFARK	(3)		(1)
		10;13	ADVFPGND**ST**ASQDVANRFARK	(3) (3)	(1) (1)	(1) (1)
		226	TIRSTLNPQWNE**S**FTFK		(2)	(2)
		319	VI**S**PSEDRK	(3) (3)	(3) (2)	(2) (3)
	**PKC Beta**	11	ADPAAGPPP**S**EGEESTVRFARK	(2)	(1)	(2)
		641	HPPVL**T**PPDQEVIR	(3)	(3)	(3)
novel	**PKC Delta**	645	ARL**S**YSDK	(3)	(3)	(3)
		647	ARLSY**S**DK	(2)	(1)	(1)
		664	NLIDSMDQSAFAGF**S**FVNPK	(1)		(3)
	**PKC Epsilon**	309	VLADLGV**T**PDK	(2)	(2)	(2)
	**PKC Theta**	685	ALIN**S**MDQNMFR	(2)	(1)	(2)
	**PKC Eta**	317	TLAGMGLQPGNI**S**PTSK	(3)	(2)	(2)
atypical	**PKC Iota**	564	PNISGEFGLDNFDSQFTNEPVQL**T**PDDDDIVRK	(3)	(1)	(3)

The number in brackets indicates in how many technical replica a given phospho-site was detected (max. 3). Each bracket provides the results of one biological experiment.

PKC mediates the phosphorylation of KCC2 and NKCC1 [48, 77, 96, 97]. The PKC family consists of 10–12 isoforms grouped into three classes [98–101]. We detected several phospho-sites in seven *hs*PKC family members (alpha, beta, delta, epsilon, theta, eta and iota; Table 4).

To summarize, according to mass spectrometry based phosphoproteome analyses, staurosporine and NEM reduce the number of detected phospho-sites of stably expressed *rn*KCC2b and endogenously expressed *hs*KCC1, *hs*KCC4, *hs*NKCC1, *hs*WNK1 and *hs*SPAK. Phosphorylation of some sites (*rn*KCC2b: Ser^31^, *hs*NKCC1:Ser^242^, *hs*KCC4: Thr^980^) was absent only after staurosporine treatment. Yet, these results can only be used as an indication since the absence of phosphorylation sites can reflect detection problems caused by low phosphorylation rates.

### Quantitative analyses of phospho-sites of *rn*KCC2b and *hs*NKCC1 upon staurosporine and NEM treatment in HEK^*rn*KCC2b^ cells

**Fig 3:**
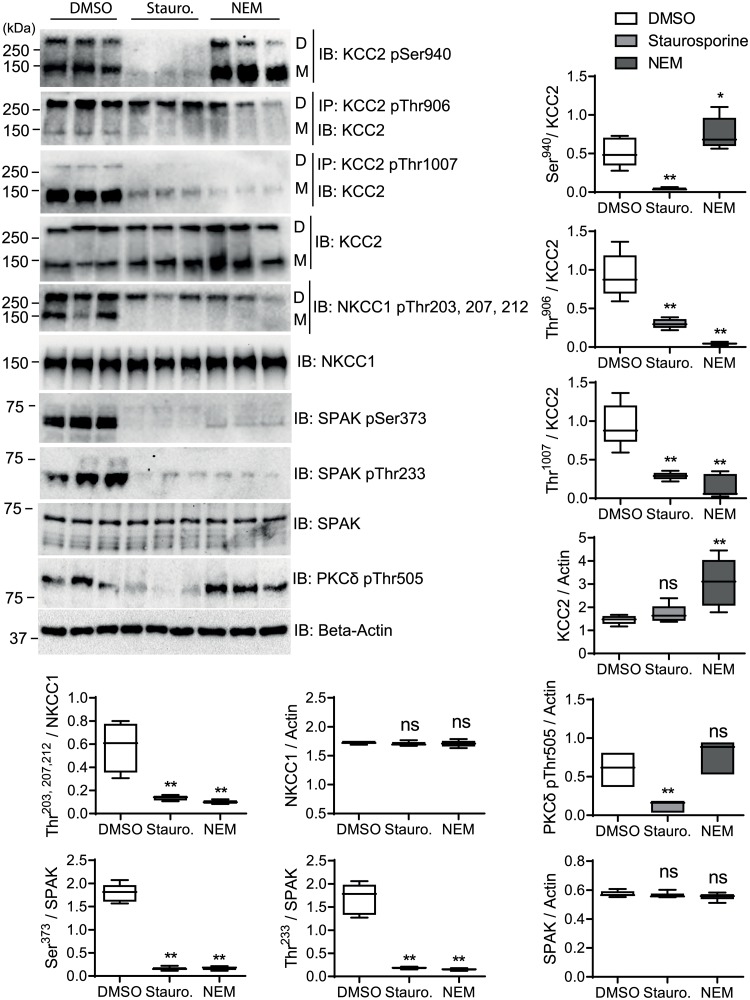
Quantitative analyses of *rn*KCC2b and *hs*NKCC1 phospho-sites upon staurosporine and NEM treatment in HEK^rnKCC2b^ cells. Stably transfected HEK^*rn*KCC2b^ cells were treated with DMSO (control), 8 μM staurosporine or 0.5 mM NEM, respectively, for 15 min. Cell lysates were harvested and subjected to immunoprecipitation (IP) and immunoblot (IB) with indicated antibodies. D, dimeric KCC2; M, monomeric KCC2. Band intensities were quantified with ImageJ software. ***, *p* < 0.001; **, *p* < 0.01; Wilcoxon-Mann-Whitney test (n = 6).

The experimental setup of our mass spectrometry-based analysis precluded quantification of changes at individual phospho-sites. We therefore applied in a next step phospho-site-specific antibody, as they were previously shown to quantitatively monitor changes in KCC2, NKCC1 and SPAK phosphorylation [23, 48, 59, 66, 70, 102]. Currently, a limited number of this class of antibodies is available for CCCs. They are directed against the well-known phospho-sites Ser^940^, Thr^906^ and Thr^1007^ in *rn*KCC2b and Thr^203, 207, 212^ in *hs*NKCC1. So far, no data are available for the staurosporine and NEM effect on Thr^906^ in *rn*KCC2b and Thr^203, 207, 212^ in *hs*NKCC1 and the staurosporine effect on Ser^940^, and Thr^1007^ in *rn*KCC2b. To examine the impact of staurosporine and NEM on the phosphorylation level of these sites, we treated HEK^*rn*KCC2^ cells with 8 μM staurosporine, 1 mM NEM or DMSO as a vehicle control for 15 min. Lysates were probed for KCC2 or NKCC1 and phosphorylation levels of each phospho-site were quantified (Fig 3). As previously described, positions Ser^940^, Thr^906^ and Thr^1007^ are phosphorylated in KCC2b, and Thr^203, 207, 212^ in NKCC1 of untreated HEK^*rn*KCC2^ cells [48, 70, 102] (Fig 3). These data corroborate the phosphoproteome analyses which revealed phosphorylation of Ser^940^, Thr^906^ and Thr^1007^ in KCC2b and Thr^212^/Thr^217^ in NKCC1 as well (Table 1).

Next, we observed the impact of staurosporine and NEM on these phospho-sites (Fig 3). Both agents decreased the phosphorylation status of the WNK/SPAK sites Thr^906^ (*p*-value for NEM or staurosporine: *p* = 0.0026) and Thr^1007^ (*p*-value for NEM or staurosporine: *p* = 0.0026) of *rn*KCC2 and Thr^203/207/212^ of *hs*NKCC1 (*p*-value for NEM and staurosporine: *p* = 0.0026) [72–75, 103]. The reduced phosphorylation of Thr^212^ in *hs*NKCC1 agrees with our phosphoproteome analyses as no phosphorylation of Thr^212^/Thr^217^ in NKCC1 was observed (Table 1).

Previous analyses showed that SPAK directly phosphorylates Thr^1007^ of *rn*KCC2 [48] and Thr^203/207/212^ of *hs*NKCC1 [70]. Treatment of HEK293 cells with NEM resulted in a decrease of phosphorylation of Thr^233^ (*p* = 0,0026), that is located in the T-loop kinase domain, and the S-loop phosphorylation site Ser^373^ of *hs*SPAK (*p* = 0.0026) (Fig 3). Both are targets of WNKs [48, 69, 93]. As no data were available for the staurosporine mediated effect, we additionally analyzed its impact on these phospho-sites in HEK293 cells. Staurosporine also reduced the phosphorylation of Thr^233^ (*p* = 0.0026) and Ser^373^ (*p* = 0.0026) in *hs*SPAK (Fig 3). Thus, both agents reduced phosphorylation levels of these SPAK phospho-sites. These data conform well to our phosphoproteomic analyses, as no phosphorylated Ser^372/373^ of *hs*SPAK was detected after treatment with either of the two agents.

Furthermore, staurosporine reduced phosphorylation of Ser^940^ (*p* = 0.0026) in HEK^*rn*KCC2b^, whereas NEM increased phosphorylation of Ser^940^ (*p* = 0.046) significantly (Fig 3). Since, Ser^940^ is directly phosphorylated by PKC [77, 104], we here analyzed the impact of both agents on the T-loop phosphorylation site Thr^505^ of PKC-δ. Autophosphorylation of this site is most probably essential for kinase activity [105–107]. Staurosporine significantly decreases Thr^505^ phosphorylation (*p* = 0.0026, Fig 3), whereas NEM slightly, but not significantly, increases Thr^505^ phosphorylation (*p* = 0.064). The different impact of both agents on the phosphorylation of Thr^505^ of PKC correlates well with their impact on Ser^940^ phosphorylation of KCC2b.

We also determined whether staurosporine or NEM altered the total protein amount of *rn*KCC2b, *hs*NKCC1 or *hs*SPAK (Fig 3). Whereas NEM resulted in increased KCC2 amount (*p* = 0.0026), no obvious change was detected upon staurosporine treatment. NKCC1 and SPAK levels were not changed significantly upon treatment with either agent.

To conclude, staurosporine and NEM reduced the phosphorylation status of SPAK (Thr^233^ and Ser^373^). This correlated with the reduction of the phosphorylation of Thr^1007^ in *rn*KCC2b and Thr^203/207/212^ in *hs*NKCC1. Additionally, both agents reduced phosphorylation of Thr^906^ in *rn*KCC2b, which is phosphorylated by WNKs and a yet unknown kinase [48]. Staurosporine also reduced phosphorylation of the PKC site Ser^940^ in *rn*KCC2b, whereas NEM increased its phosphorylation level. This correlated with the reduction of Thr^505^ phosphorylation of PKC-δ upon staurosporine treatment and the impact of NEM to increase Thr^505^ phosphorylation.

### Quantitative analyses of phospho-sites of KCC2 and NKCC1 upon staurosporine or NEM treatment of rat immature hippocampal neurons

**Fig 4:**
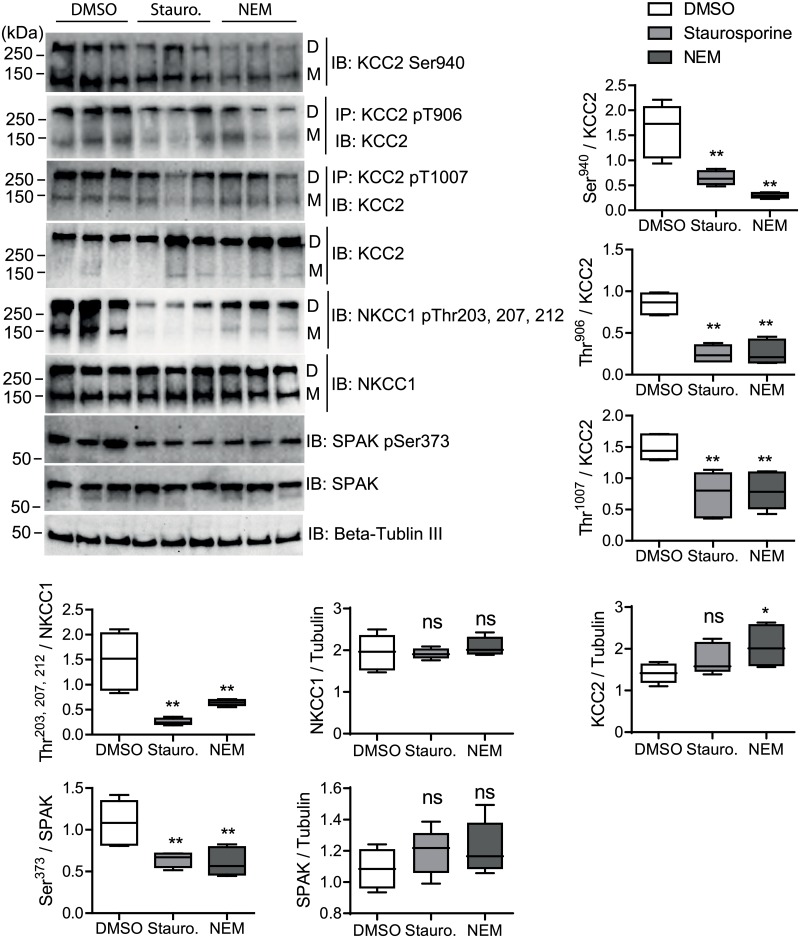
Quantitative analyses of *rn*KCC2 and *rn*NKCC1 phospho-sites upon staurosporine or NEM treatment in immature hippocampal neurons. Immature rat hippocampal neurons were treated with DMSO (control), 8 μM staurosporine or 0.5 mM NEM, respectively, for 15 min. Neuronal lysates were harvested and subjected to immunoprecipitation (IP) and immunoblot (IB) with the indicated antibodies. D, dimeric KCC2; M, monomeric KCC2. Band intensities were quantified using ImageJ software. ***, *p* < 0.001; **, *p* < 0.01; Wilcoxon-Mann-Whitney test (n = 6).

As KCC2 is predominantly expressed in neurons [20], we analyzed for the first time the impact of staurosporine and NEM on the phosphorylation of specific phospho-sites of endogenously expressed KCC2 and NKCC1 using immature (9 DIV) primary rat hippocampal neurons (Fig 4). At this age cultured hippocampal neurons exhibit prominent level of Thr^906^ and Thr^1007^ KCC2 phosphorylation [66] that could be a subject of modulation by staurosporine and NEM. To this end, we treated neurons with 8 μM staurosporine, 0.5 mM NEM or DMSO as a vehicle control for 15 min. NEM was reduced to 0.5 mM, since higher concentrations induced cell death. As described for the analyses in stably transfected HEK^*rn*KCC2b^ cells, we used phospho-specific KCC2 and NKCC1 antibodies to quantify phosphorylation levels of each phospho-site relatively to the DMSO control. In untreated cultured immature hippocampal neurons, Ser^940^, Thr^906^ and Thr^1007^ in KCC2 and Thr^203, 207, 212^ in NKCC1 were phosphorylated, similar to stably transfected HEK^*rn*KCC2b^ cells (Fig 4).

Most actions of staurosporine and NEM, as monitored by immunblots, were similar between HEK^*rn*KCC2b^ cells and immature hippocampal neurons. Both agents resulted in decreased phosphorylation of Thr^1007^ in KCC2 (*p*-value for NEM and staurosporine: *p* = 0.0026), Thr^203/207/212^ in NKCC1 (*p*-value for NEM and staurosporine: *p* = 0.0026), and Ser^373^ in SPAK (*p*-value for NEM: *p* = 0.0026 and staurosporine: *p* = 0.0026). Additionally, both agents reduced phosphorylation of the WNK-dependent phospho-site Thr^906^ in KCC2 (*p*-value for NEM and staurosporine: *p* = 0.0026). Finally, NEM increased the total protein level of KCC2, as observed in HEK293 cells (*p* = 0.015) (Fig 4). We were not able to detect the phosphorylation of Thr^233^ of SPAK using phospho-specific antibodies.

A marked difference, however, was observed for the PKC dependent phospho-site Ser^940^. Here, treatment with NEM reduced phosphorylation of Ser^940^ (*p* = 0.0026) (Fig 4), contrary to the results obtained in stably transfected HEK^*rn*KCC2b^ cells (Fig 3). Treatment with staurosporine also resulted in reduced phosphorylation of Ser^940^ (*p* = 0.0026), which was similar to its action in HEK^*rn*KCC2b^ cells. We could not detect Thr^505^ phosphorylation of PKC-δ due to low expression rates.

To sum up, NEM affected the phosphorylation status of Ser^940^ in immature hippocampal neurons (decrease) in the opposite way compared to HEK^*rn*KCC2b^ cells (increase). All other effects of staurosporine and NEM were similar between immature hippocampal neurons and HEK293 cells, i.e. both reduced the phosphorylation status of Ser^940^, Thr^906^, and Thr^1007^ in KCC2 and Thr^203/207/212^ in *hs*NKCC1.

## Discussion

**Fig 5:**
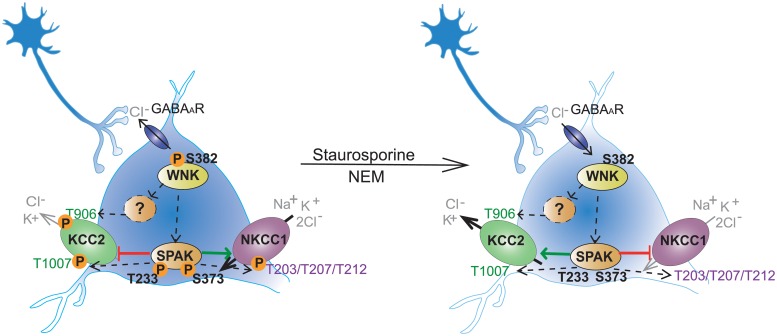
Staurosporine and NEM impair the WNK-SPAK/OSR1 mediated phosphorylation of KCC2 and NKCC1. In immature hippocampal neurons, the WNK mediated phosphorylation of SPAK/OSR1 directly phosphorylates T^1007^ of KCC2 and T^203^, T^207^ and T^212^ of NKCC1. WNKs also interact with a yet unknown kinase to phosphorylate Thr^906^ of KCC2. Phosphorylation of these residues decreases KCC2 and increases NKCC1 activity. Application of staurosporine or NEM decreases phosphorylation of S^382^ of WNK1 and T^233^ and S^373^ of SPAK. This likely results in reduction of phosphorylation of T^906^ and T^1007^ in KCC2 and of T^203^, T^207^ and T^212^ in NKCC1. Dephosphorylation of these residues increases KCC2 activity and decreases NKCC1 activity. The figure is modified from Moore et al. [39].

KCC2 and NKCC1 are key players for the development and maintenance of fast inhibitory neurotransmission. Their oppositely directed transport of K^+^ and Cl^-^ ions within the same neuronal population necessitates a precisely coordinated regulatory mechanism for efficient setting of the intracellular [Cl^-^] concentration [4, 38, 55–60] (Fig 5).

Posttranslational regulation via the WNK-SPAK/OSR1 dependent phosphorylation represents a potent mechanism to regulate transport activity of KCC2 and NKCC1 in a reciprocal way [60, 108–110] (Fig 5). Here, we show that staurosporine and NEM decrease phosphorylation of Thr^233^ and Ser^373^ in SPAK, of Thr^1007^ in *rn*KCC2 and Thr^203^, Thr^207^ and Thr^212^ in *hs*NKCC1 in both HEK293 cells and immature cultured hippocampal neurons. Since SPAK directly impairs phosphorylation of Thr^1007^ in *rn*KCC2 and Thr^203^, Thr^207^ and Thr^212^ in *hs*NKCC1 [38, 48, 59, 60, 63, 68–76], our data suggest that staurosporine and NEM directly affect the WNK-SPAK/OSR1 mediated phosphorylation of these residues in KCC2 and NKCC1. The data are in line with previous analyses showing that NEM reduces phosphorylation of Ser^373^ in SPAK and Thr^1007^ in KCC2 using HEK293 cells and immature cortical neurons [48]. Furthermore, application of staurosporine and NEM decreases phosphorylation of Thr^906^ in *rn*KCC2. This site is directly phosphorylated by WNKs and a yet unknown kinase [48]. However, functional in-depth analyses such as mutagenic approaches are required to prove a causal relation between dephosphorylation of SPAK Thr^233^ and Ser^373^ and dephosphorylation of the specific KCC2 and NKCC1 phospho-sites upon staurosporine and NEM treatment.

Recent analyses demonstrated that dephosphorylation of Thr^906^ and Thr^1007^ increases KCC2 activity [61, 64–66], whereas dephosphorylation of Thr^203^, Thr^207^ and Thr^212^ decreases NKCC1 activity [45, 51]. Furthermore, staurosporine and NEM results in activation of KCC2 (this study and [40, 47, 48, 59, 60, 79, 80], whereas they reduce NKCC1 activity [43–46, 51]. This suggests, that staurosporine and NEM mediated dephosphorylation of these phospho-sites result in a reciprocal regulation of KCC2 (activation) and NKCC1 (inactivation) activity most likely via the WNK/SPAK-dependent phosphorylation pathway. This is also in line with the observation in immature hippocampal neurons that KCC2 can rapidly be activated by staurosporine [42].

Another key regulatory KCC2 phospho-site is the PKC-mediated phosphorylation of Ser^940^. Phosphorylation of Ser^940^ enhances KCC2 cell surface expression and increases ion transport activity, whereas mutation of serine to alanine (mimicking the dephosphorylated state) results in transport activity that is equal or decreased compared to wild-type KCC2 (KCC2^wt^) [65, 77, 111]. Our data demonstrate that staurosporine and NEM can differentially affect Ser^940^ phosphorylation. Treatment of immature hippocampal neurons with either agent results in decreased phosphorylation of Ser^940^. However, treatment of HEK^*rn*KCC2b^ cells with staurosporine decreases, whereas NEM increases phosphorylation of Ser^940^. This is in line with the different effect of these agents on transport activity of the phosphomutants Ser^31A/D^, Thr^34A/D^, Thr^999A^ and Thr^1008A/D^ [40].

Moreover, NEM has a cell-type specific impact on Ser^940^ phosphorylation. In immature cortical neurons [48] and HEK293 cells, NEM increases Ser^940^ phosphorylation, whereas it decreases Ser^940^ phosphorylation in immature cultured hippocampal neurons (Fig 4). The mechanisms that cause this opposite effect of NEM on Ser^940^ phosphorylation in different tissues is unclear. One possibility is that different NEM concentrations (HEK293 cells: 1 mM and 0.1 mM; immature hippocampal neurons: 0.5 mM; immature cortical neurons: 0.1 mM [48]) affect different regulatory pathways. Alternatively, tissues-specific sets of PKC isoforms and phosphatases result in different phosphorylation patterns. Indeed, the PKC family consists of 10–12 isoforms grouped into three classes [98–101] that vary in their expression profile [112] and regulation of their activity through several regulatory proteins, co-factors and second messenger cascades [100, 101, 113]. This offers the opportunity to differentially regulate KCC2 function in distinct neuronal populations through PKC. In HEK^*rn*KCC2b^ cells, we showed that staurosporine reduced and NEM slightly increased phosphorylation of the T-loop phospho-site Thr^505^ of PKC-δ. This correlated with decreased KCC2 Ser^940^ phosphorylation upon staurosporine treatment and increased phosphorylation of this residue upon NEM treatment. This suggests, that both agents directly act on PKC-δ mediating the phosphorylation of Ser^940^ in HEK^*rn*KCC2b^ cells. However, more functional in-depth analyses are required to elucidate the causal link between dephosphorylation of PKC-δ Thr^505^ and dephosphorylation of KCC2 Ser^940^ upon staurosporine treatment. Since we were unable to detect phosphorylation of PKC-δ Thr^505^ in immature hippocampal neurons, we suggest that other PKC isoforms are involved in the direct phosphorylation of KCC2 Ser^940^ in immature hippocampal neurons.

Our data furthermore reveal that staurosporine (HEK^*rn*KCC2b^ cells, immature hippocampal neurons) and NEM treatment (immature hippocampal neurons) decrease Ser^940^ phosphorylation resulting in an equal or diminished transport activity compared to KCC2^wt^ [65, 77, 111]. We therefore conclude that Ser^940^ is not the key regulatory phospho-site mediating the staurosporine and NEM-based stimulation effect on KCC2. This is in line with recent published analyses showing that NEM still enhances the transport activity of Ser^940A^ (mimicking dephosphorylated state), indicating that other phospho-sites are important in NEM-dependent stimulation [40, 48].

NEM but not staurosporine increased total KCC2 amount in HEK293 cells and immature cultured hippocampal neurons. In immature cortical neurons, Deep and coworkers [48] detected the same trend of enhanced total KCC2 abundance (albeit not significant), resulting in increased cell surface expression [48]. This suggests that in contrast to staurosporine, NEM increases KCC2 expression and trafficking that could result in a higher KCC2 activity. The different impact of NEM on total KCC2 abundance in HEK293 in the study of Deep and coworkers (no increase) and our analyses (increase) could result from different NEM concentration used in the experiments (0.1 mM vs. 1 mM).

Via mass spectrometry analysis, we identified several new phosphorylation sites whose function awaits further investigation. These sites are the N-terminal KCC2 phospho-site Thr^32^, and the C-terminal NKCC1 phospho-sites Ser^242^, Thr^266^, Th^268^, Ser^940^, Tyr^956^ and Ser^957^. Future studies should investigate their regulatory impact on KCC2 and NKCC1 activity.

## Conclusions

In conclusion, our data identify molecular mechanisms involved in staurosporine and NEM mediated changes in transport activity of KCC2 and NKCC1, which are a defining feature of CCCs [114]. The observation of cell-type specific action of these agents is in line with different reversal potentials in mature neuronal populations [115] and calls for comprehensive neuron subtype-specific phospho analysis. The recently reported structural data of CCCs [116–119] finally lay the foundation to analyze jointly the physiological role of phosphorylation and underlying structural changes to obtain an integrative and mechanistic view of the action of phosphorylation.

## Supporting information

S1 Table Phospho-sites in PhosphoSitePlus detected by mass spectrometry analyses. (DOCX)

S2 Table Phospho-sites in PhosphoSitePlus detected by mass spectrometry analyses. (DOCX)

S3 Table Phospho-sites in PhosphoSitePlus detected by mass spectrometry analyses. (DOCX)

S1 Fig Quantitative analyses of *rn*KCC2 and *hs*NKCC1 phospho-sites upon staurosporine and NEM treatment in HEK^rnKCC2b^ cells. (PDF)

S2 Fig Quantitative analyses of *rn*KCC2 and *hs*NKCC1 phospho-sites upon staurosporine and NEM treatment in immature hippocampal neurons. (PDF)
