# Supplementary material for: Staurosporine and NEM mainly impair WNK-SPAK/OSR1 mediated phosphorylation of KCC2 and NKCC1
Source: PLoS One. 2020 May 15;15(5):e0232967. doi: 10.1371/journal.pone.0232967 (PMC7228128; doi:10.1371/journal.pone.0232967)
Supplement: S1 Table — (DOCX) [file pone.0232967.s001.docx]

**Suppl. Table 1:**

**Phospho-sites in PhosphoSitePlus detected by mass spectrometry analyses**

| **Phosphosite**  **Plus *hs*NKCC1** | **Transport activity measured by:** |
| --- | --- |
| S^23^ |  |
| T^75^ |  |
| S^77^ | ([105](#_ENREF_105),[106](#_ENREF_106)) |
| S^79^ |  |
| S^123^ |  |
| S^132^ |  |
| T^203^ | ([71](#_ENREF_71),[72](#_ENREF_72),[107](#_ENREF_107)) |
| T^207^ | ([71](#_ENREF_71),[72](#_ENREF_72),[107](#_ENREF_107)) |
| Y^208^ |  |
| T^212^ | ([71](#_ENREF_71),[72](#_ENREF_72),[107](#_ENREF_107)) |
| **T^217^** | ([72](#_ENREF_72)) |
| **Y^227^** |  |
| T^230^ | ([72](#_ENREF_72)) |
| **S^242^** | ([106](#_ENREF_106)) |
| S^265^ |  |
| **T^266^** |  |
| **T^268^** |  |
| Y^275^ |  |
| T^276^ |  |
| Y^353^ |  |
| Y^354^ |  |
| S^836^ |  |
| Y^842^ |  |
| Y^855^ |  |
| **S^940^** |  |
| S^944^ |  |
| T^947^ |  |
| S^953^ |  |
| **Y^956^** |  |
| **S^957^** |  |
| T^984^ |  |
| S^994^ |  |
| S^1012^ |  |
| Y^1211^ |  |
| S^1212^ |  |

Abbreviations used are as follows: *hs*, *homo sapiens*. Phospho-sites that were detected in the present mass spectrometry study are marked in bold.
