# Supplementary material for: Staurosporine and NEM mainly impair WNK-SPAK/OSR1 mediated phosphorylation of KCC2 and NKCC1
Source: PLoS One. 2020 May 15;15(5):e0232967. doi: 10.1371/journal.pone.0232967 (PMC7228128; doi:10.1371/journal.pone.0232967)
Supplement: S2 Table — (DOCX) [file pone.0232967.s002.docx]

**Suppl. Table 2:**

**Phospho-sites in PhosphoSitePlus detected by mass spectrometry analyses**

| **Phosphosite**  **Plus *hs*KCC1** | **Phosida**  ***hs*KCC1** | **Transport activity measured by:** |
| --- | --- | --- |
| Y17 |  |  |
| S24 |  |  |
| Y28 |  |  |
| S37 | S37 |  |
| S46 | S46 |  |
| S47 |  |  |
| S51 | S51 |  |
| S56 |  |  |
| Y61 |  |  |
| Y62 |  |  |
| S8 |  |  |
| Y89 |  |  |
| S104 |  |  |
| T269 |  |  |
| T263 |  |  |
| S407 |  |  |
| Y411 |  |  |
| T418 |  |  |
| S441 |  |  |
| T725 |  |  |
| S863 |  |  |
| T926 | T926 |  |
| S958 |  |  |
| S964 |  |  |
| Y966 |  |  |
| S967 | S967 |  |
| S973 |  |  |
| **T983** |  | ([60](#_ENREF_60)) |
| T1021 |  |  |

Abbreviations used are as follows: *hs*, *homo sapiens; rn*, *Rattus norvegicus*; *mm*, *Mus musculus*. Phospho-sites that were detected in the present mass spectrometry study are marked in bold.
