# Supplementary material for: Staurosporine and NEM mainly impair WNK-SPAK/OSR1 mediated phosphorylation of KCC2 and NKCC1
Source: PLoS One. 2020 May 15;15(5):e0232967. doi: 10.1371/journal.pone.0232967 (PMC7228128; doi:10.1371/journal.pone.0232967)
Supplement: S3 Table — (DOCX) [file pone.0232967.s003.docx]

**Suppl. Table 3:**

**Phospho-sites in PhosphoSitePlus detected by mass spectrometry analyses**

| **Phosphosite**  **Plus *hs*KCC4** | **Phosida**  ***hs*KCC4** | **Transport activity measured by:** |
| --- | --- | --- |
| T25 |  |  |
| T30 |  |  |
| S40 |  |  |
| S50 |  |  |
| S62 |  |  |
| S78 |  |  |
| Y92 |  |  |
| T93 |  |  |
| S96 |  |  |
| S108 |  |  |
| S669 |  |  |
| T715 |  |  |
| S716 |  |  |
| Y735 |  |  |
| **T926** | **T926** | ([108](#_ENREF_108)) |
| S960 |  |  |
| T968 |  |  |
| T973 |  | ([60](#_ENREF_60)) |
| **T980** |  | ([60](#_ENREF_60),[108](#_ENREF_108)) |
| T982 |  | ([60](#_ENREF_60)) |
| Y991 |  |  |
| T996 |  |  |
| S997 |  |  |
| S1006 |  |  |
| S1012 |  |  |

Abbreviations used are as follows: *hs*, *homo sapiens*; *rn*, *Rattus norvegicus*; *mm*, *Mus musculus.* Phospho-sites that were detected in the present mass spectrometry study are marked in bold.
