# Supplementary material for: Staurosporine and NEM mainly impair WNK-SPAK/OSR1 mediated phosphorylation of KCC2 and NKCC1
Source: PLoS One. 2020 May 15;15(5):e0232967. doi: 10.1371/journal.pone.0232967 (PMC7228128; doi:10.1371/journal.pone.0232967)

Supplementary Figure S1 for Figure 3: Quantitative analyses of *rn*KCC2 and *hs*NKCC1 phospho-sites upon staurosporine and NEM treatment in HEK^{rnKCC2b} cells

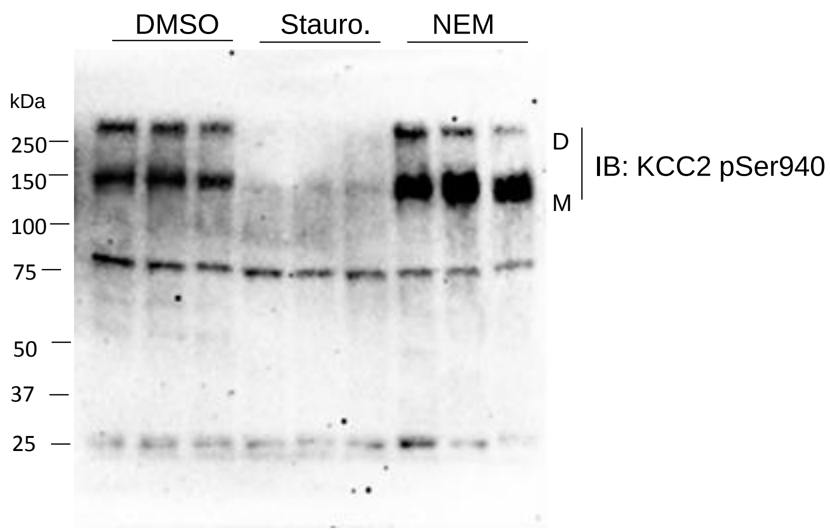

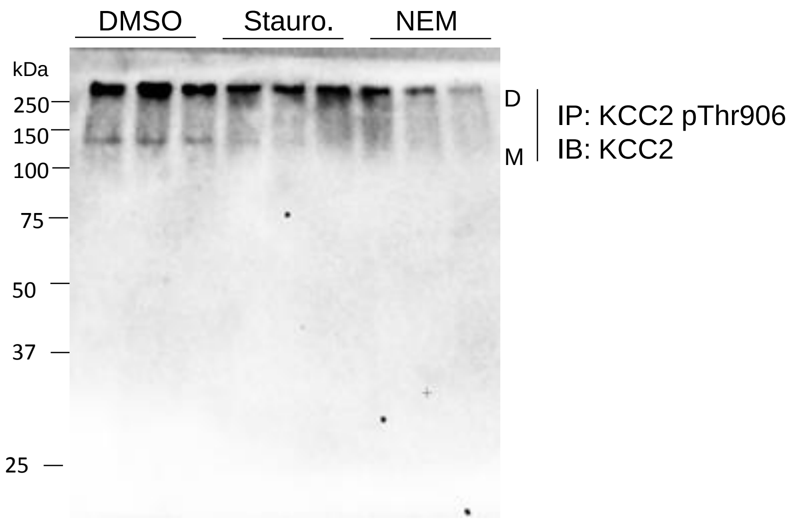

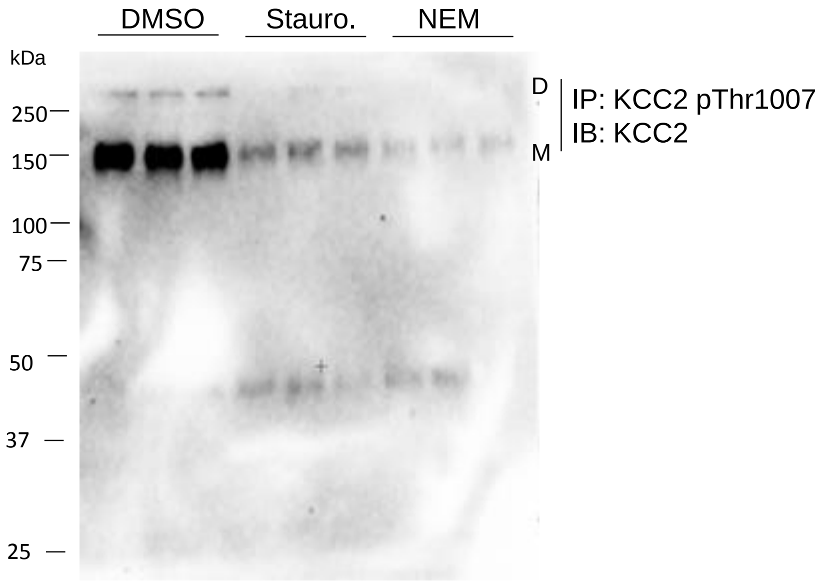

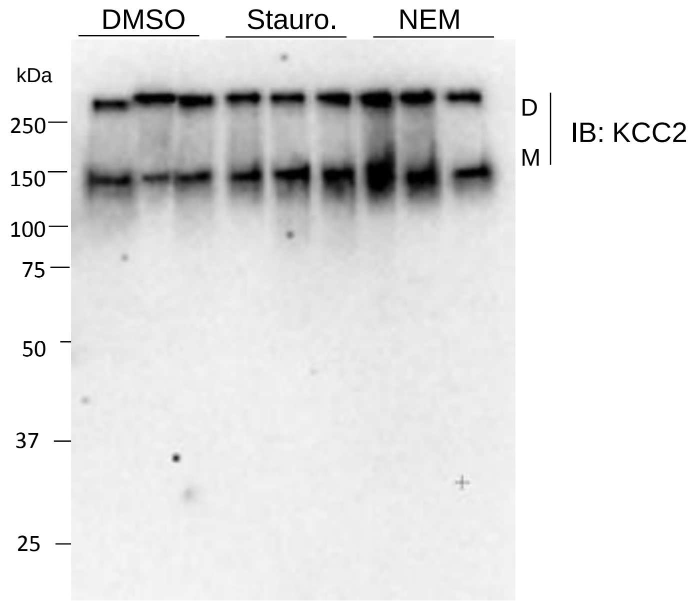

Supplementary Figure S1 for Figure 3: Quantitative analyses of *rn*KCC2 and *hs*NKCC1 phospho-sites upon staurosporine and NEM treatment in HEK^{rnKCC2b} cells

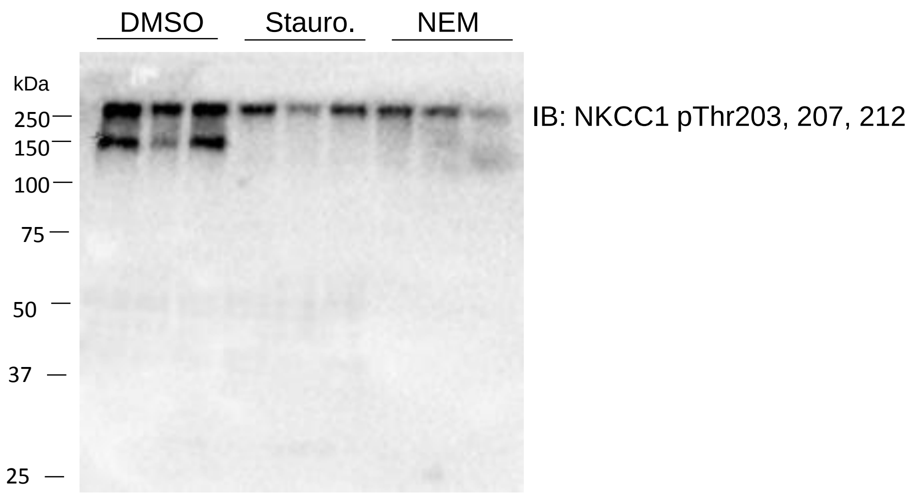

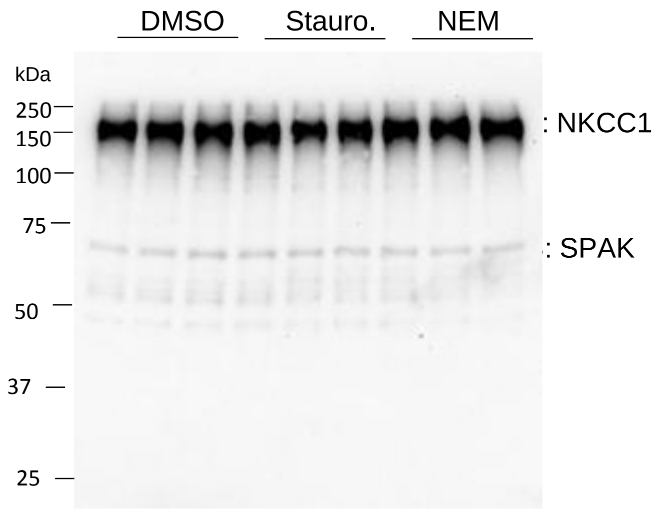

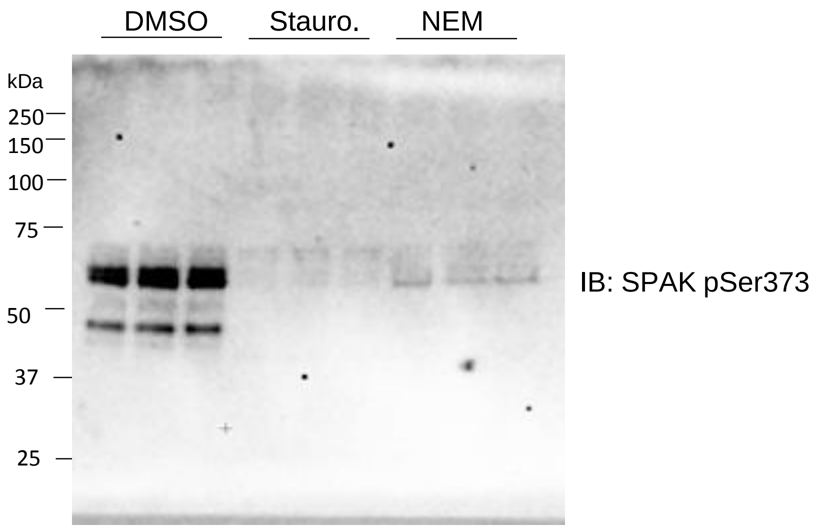

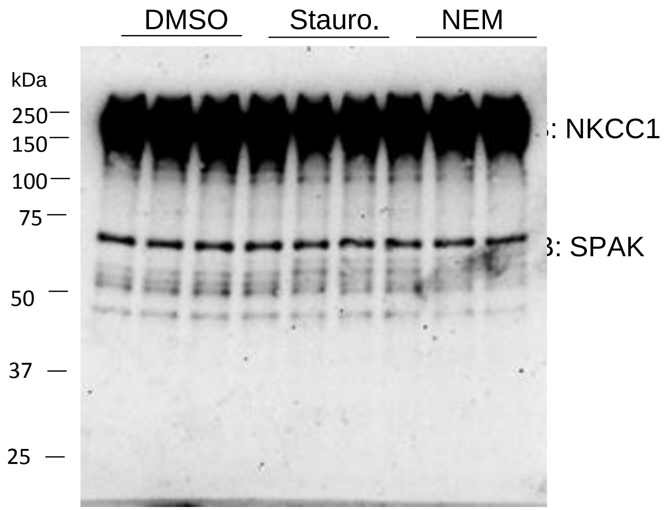

Supplementary Figure S1 for Figure 3: Quantitative analyses of *rn*KCC2 and *hs*NKCC1 phospho-sites upon staurosporine and NEM treatment in HEK^{rnKCC2b} cells

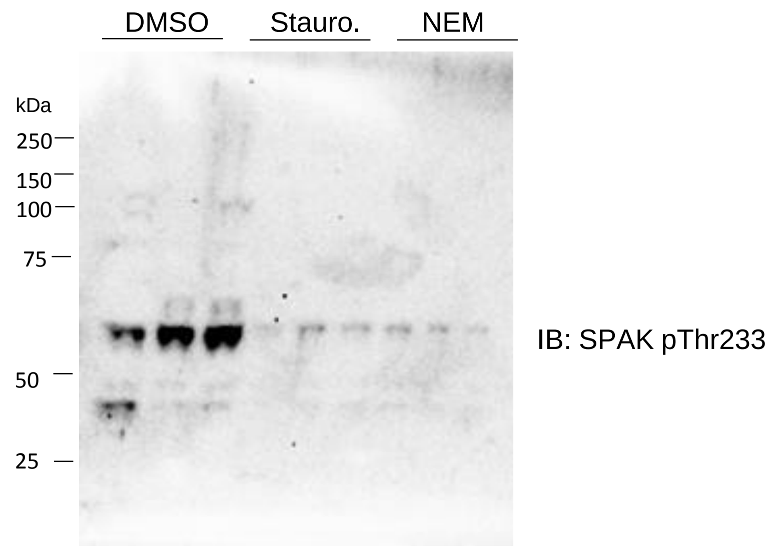

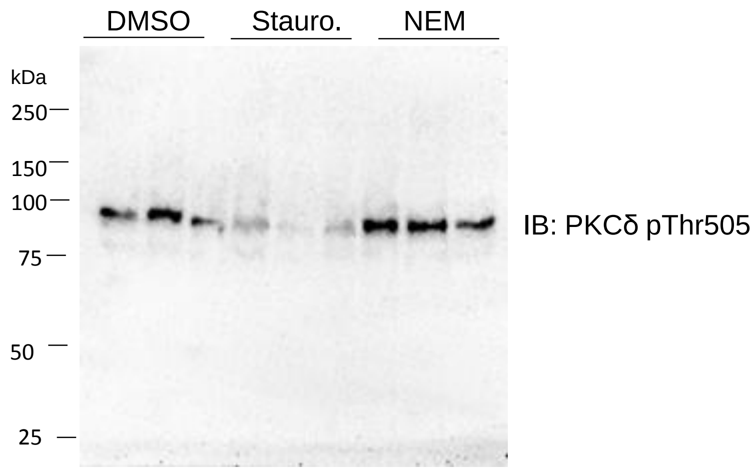

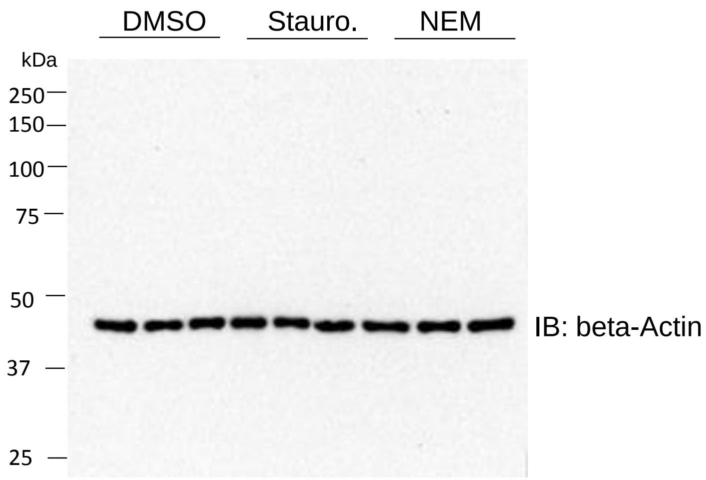

Supplement: S1 Fig — (PDF) [file pone.0232967.s004.pdf]
