# Supplementary material for: Staurosporine and NEM mainly impair WNK-SPAK/OSR1 mediated phosphorylation of KCC2 and NKCC1
Source: PLoS One. 2020 May 15;15(5):e0232967. doi: 10.1371/journal.pone.0232967 (PMC7228128; doi:10.1371/journal.pone.0232967)

Supplementary Figure S2 for Figure 4: Quantitative analyses of *rn*KCC2 and *hs*NKCC1 phospho-sites upon staurosporine and NEM treatment in immature hippocampal neurons

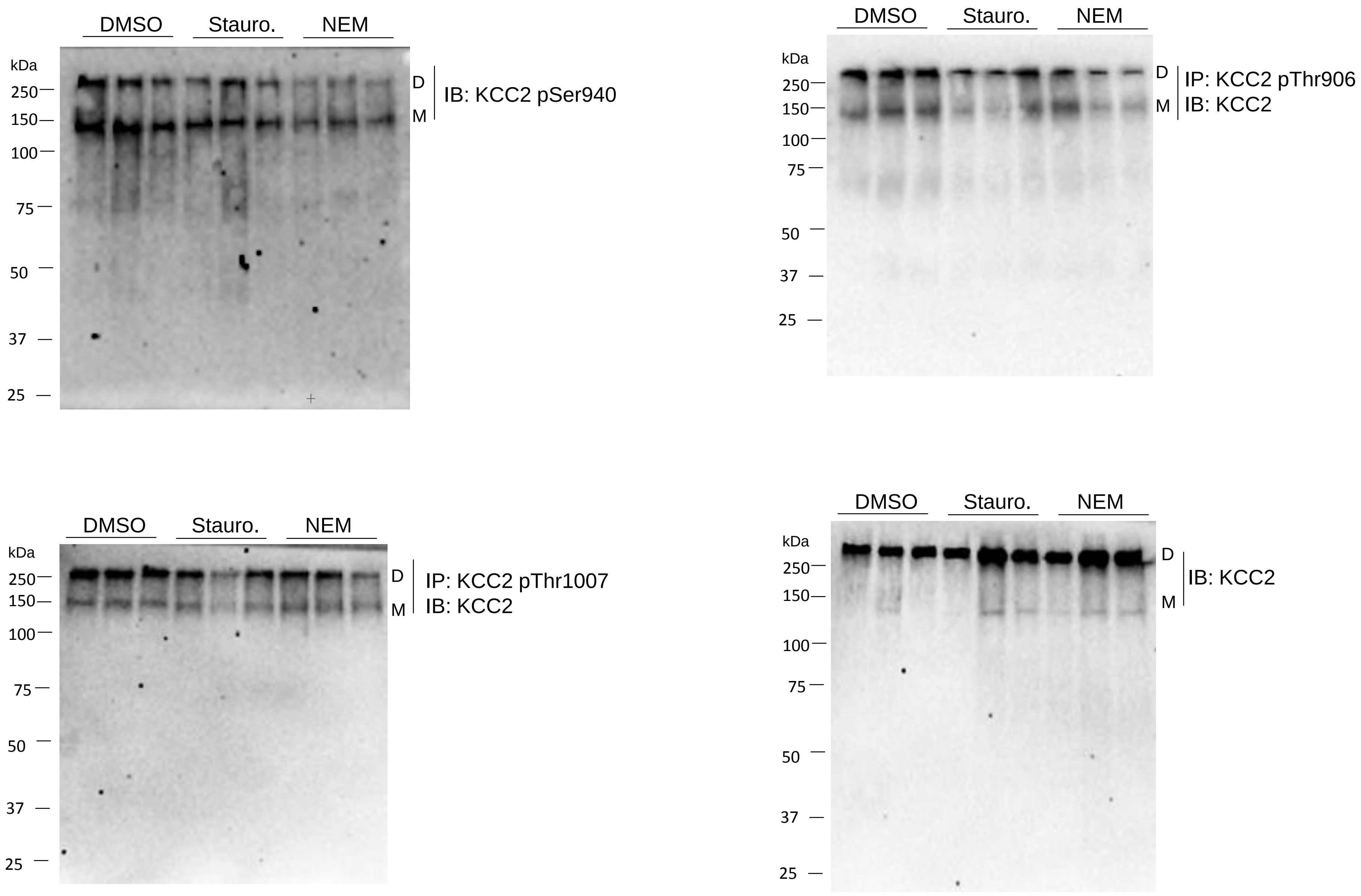

Supplementary Figure S2 for Figure 4: Quantitative analyses of *rn*KCC2 and *hs*NKCC1 phospho-sites upon staurosporine and NEM treatment in immature hippocampal neurons

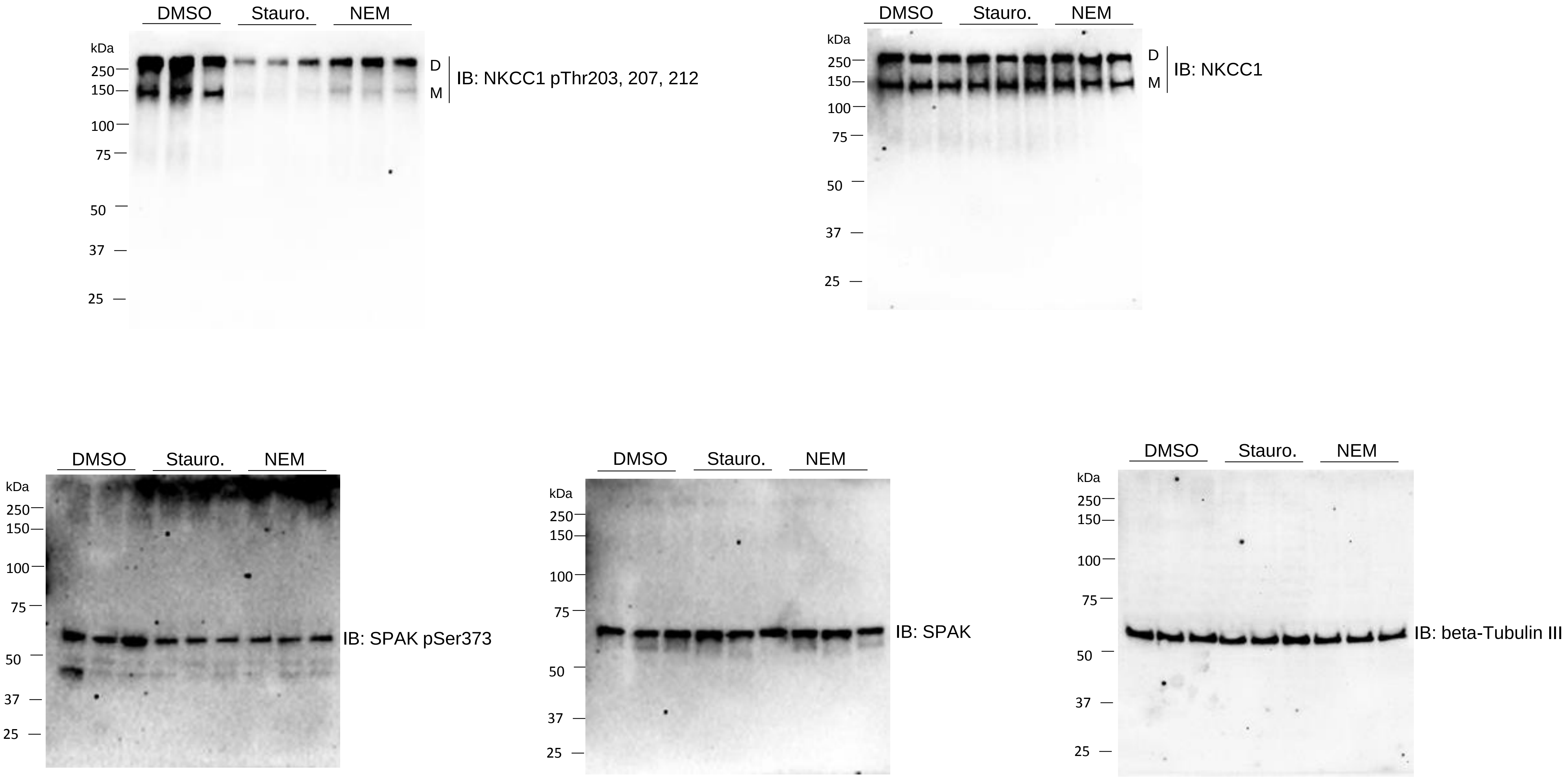

Supplement: S2 Fig — (PDF) [file pone.0232967.s005.pdf]
